# Medical students’ affective reactions to workplace experiences: qualitative investigation in a Chinese cultural context

**DOI:** 10.1186/s12909-020-02335-7

**Published:** 2020-11-04

**Authors:** Huei-Ming Yeh, Wan-Hsi Chien, Daniel Fu-Chang Tsai, Tim Dornan, Ling-Ping Lai, Chun-Lin Chu

**Affiliations:** 1 Department of Anesthesiology, National Taiwan University Hospital, Taipei, Taiwan; 2 Department of Psychiatry, Taipei City Psychiatric Center, Taipei City Hospital, Taipei, Taiwan; 3 Research Institute of Medical Education & Bioethics, National Taiwan University College of Medicine, and Attending Physician, Department of Medical Research, National Taiwan University Hospital, Taipei, Taiwan; 4 Centre for Medical Education, Queen’s University Belfast, Belfast, UK; 5 Maastricht University, Maastricht, The Netherlands; 6 Department of Cardiology, National Taiwan University Hospital, Taipei, Taiwan; 7 Department of Anesthesiology, National Taiwan University Hospital Yun-Lin Branch, No 579, Sec 2 Yun-Lin Road, Douliu, YunLin Taiwan, ROC

**Keywords:** Affective reaction, Workplace experience, Cultural hegemony, Oral diary

## Abstract

**Background:**

Compassion fatigue, unprofessional behavior, and burnout are prompting educators to examine medical students’ affective reactions to workplace experiences. Attributes of both students and learning environments are influenced by their socio-cultural backgrounds. To prevent ‘educational cultural hegemony’, opinion leaders have advocated research in under-represented cultural contexts, of which Asia is a prime example. This study aimed to broaden the discourse of medical education by answering the question: how do students react affectively to workplace experiences in a Chinese cultural context?

**Methods:**

In 2014, the authors recruited five female and seven male Taiwanese clerkship students to make 1–2 audio-diary recordings per week for 12 weeks describing affective experiences, to which they had consciously reacted. The authors analyzed transcripts of these recordings thematically in the original Mandarin and prepared a thick description of their findings, including illustrative extracts. An English-speaking education researcher helped them translate this into English, constantly comparing the interpretation with the original, untranslated data.

**Results:**

(Mis) matches between their visions of future professional life and clerkship experiences influenced participants’ affective reactions, thoughts, and behaviors. Participants managed these reactions by drawing on a range of personal and social resources, which influenced the valence, strength, and nature of their reactions. This complex set of interrelationships was influenced by culturally determined values and norms, of which this report provides a thick description.

**Conclusion:**

To avoid educational cultural hegemony, educators need to understand professional behavior in terms of complex interactions between culturally-specific attributes of individual students and learning environments.

**Trial registration:**

The ethics committee of the National Taiwan University (NTU) Hospital gave research ethics approval (20130864RINB).

## Background

Medical educators have been criticized for paying insufficient attention to doctors’ emotions [1]. Being a doctor arouses emotions because shouldering responsibility for patients’ well-being, recovery, and survival is hard work. Physicians reap rich rewards when patients get better and are satisfied but not all patients get better and even those who do get better may not be satisfied. The death of patients, in particular, can make doctors feel anger, helplessness, guilt, and pain [2]. Emotions aroused by clinical practice are important because they impact on physicians’ relationships with patients [1, 3]. Unresolved negative emotions, moreover, can result in physicians developing ‘empathy fatigue’, poor psychological wellbeing and, ultimately, burnout [1]. The need to maintain an emotionally healthy workforce with high professional standards makes it important to pay attention to the emotional effects of medical practice and for educators to identify ways of preventing or mitigating negative effects.

In undergraduate curricula, researchers have examined medical students’ emotional reactions to a range of experiences. Emotions are at their most intense when students first interact with patients, particularly very sick ones [4–6]. These emotions include anxiety, helplessness, role confusion, and guilt [7]. Students’ may experience an instinctive emotional concern about patients’ suffering, which pulls against a perceived need to demonstrate professional detachment [8]. The complex response to tensions such as these is influenced by intra-personal and cultural factors [8, 9]. Some students regulate their emotions by distracting themselves, focusing on the task rather than the patient, and/or suppressing emotions altogether; others may not be able to regulate their emotions at all [10]. Unregulated negative emotions can result in so-called compassion fatigue, a decline in empathy, and an adverse effect on students’ health and professional behavior [1]. Observing that medical educators tends to address the emotional realm by ignoring, detaching from, or distancing emotions, Shapiro et al. [1] proposed that educators should help students pay more attention to the emotions of themselves and patients during medical training.

The relationship between students’ mood, motivation, confidence, and ‘other-directed’ emotions and their development of professional identity is a focus of attention in medical education research [5, 11–14]. Powerful emotional reactions, particularly those linked to personal values, strongly influence students’ identity formation and the reverse is also true: identity formation arouses strong emotions linked to students’ sense of self and legitimacy in practice settings [13, 14]. This set of emotional reactions, which includes mood, moral emotions, and achievement emotions and extends beyond these to include values and identity (encompassed by the term ‘affects’) is an important component of medical professionalism [14].

Educators have examined ways of helping students develop this aspect of professionalism. They have devised and piloted curricula to support students’ affective development [15]. Supported reflection in Balint Groups has helped students explore their affective reactions, and how these affect relationships with patients [16]. Emotional intelligence training has been a particular focus of attention because this has the potential to help students monitor their own emotional states, discriminate between different emotions, and use emotional information to guide their thinking and behavior [17–19]. Emotional intelligence training has shown some promise, but effect sizes have been modest and inconsistent [19, 20].

Given the importance of students’ affective development and the lack of adequate theoretical models to guide faculty in addressing it [1], the authors systematically reviewed articles published in the years 2007–2018 inclusive, to identify trustworthy research that showed how curriculum interventions influenced medical students’ affective reactions [14]. The substantial body of evidence that exists but is slanted towards English-language and European countries: 43% of 70 empirical articles came from Europe, 39% from the USA and Canada, 13% from Australia and New Zealand, 4% from Africa, and just 1% from Asia [14]. Since the scholarship of medical education is regarded, increasingly, as a trans-national enterprise [21], our knowledge of the causes of medical students’ affective reactions remains incomplete.

There is evidence that affective responses to workplace experiences, and the effects of these on professional identity development, differ significantly between cultures [9, 21]. Our earlier publication [9] described how Chinese morality, informed by Confucian values, strongly emphasises social relationships, whereas western moral systems, informed by Judeo-Christian traditions, emphasise the autonomy of individuals. The ideal of a ‘good doctor’, in Chinese contexts, includes moral self-cultivation, social-political welfare promotion, humaneness, and behaving righteously. Comparing Taiwanese and Dutch medical students, we found that the former described their affective reactions within a rich social discourse, where younger people looked up to their seniors and patients were regarded somewhat paternalistically. The affective reactions of Dutch students, in contrast, were closely linked to the development of practical abilities and clinical skills. Dutch students related to a small number of influential teachers, whom they were prepared to criticize freely, framed patients as vulnerable and in need of medical help, and were quite strongly focused on personal achievement [9, 21].

This brief review confirms that affective reactions to workplace experiences play a significant part in the development of future doctors’ professional identities and, as a result, their medical professionalism. Opinion-leaders have provided evidence that non-Western populations could increase our understanding of affective reactions, identity development, and the development of professionalism and called on researchers to broaden the professional identity formation discourse to include non-Western approaches and notions [9, 21]. Despite that, a large proportion of the world’s medical students are under-represented in the existing evidence-base [14]. To prevent ‘educational cultural hegemony’ (Western researchers presenting an authoritative definition of reality, which marginalizes cultures that are less well represented by research evidence [22]), we need to widen our knowledge of Asian students’ affective reactions to workplace experiences. Our aim was to broaden the discourse of workplace medical education by answering the question: how do students react affectively to workplace experiences in a Chinese cultural context?

## Methods

### Ethics approval

The ethics committee of the National Taiwan University (NTU) Hospital gave research ethics approval (20130864RINB).

### Setting

The setting was a 7-year undergraduate program in the National Taiwan University (NTU) Hospital, Taipei. The first four years are non-clinical. In Year 5, students have their first contact with patients, most of which consists of observation rather than active involvement in delivering patient care. In Year 6, students spend complete working days in the hospital and take supervised responsibility for 1–2 patients at a time. Year 7 consists of internships, where students take primary responsibility for patient care, including performing basic clinical procedures.

### Participants, recruitment, and consent

Participants were medical students in the sixth academic year, who had entered clerkships two months previously and were rotating monthly between a wide range of specialties. We chose students at this stage because they had relatively recently entered clerkships and were under pressure to acquire competences that would allow them to fulfill their daily duties and cope with a heavy clinical workload in the following year’s internships. We expected these conditions to heighten their affective reactions. We posted an invitation in classrooms and on the students’ website. The notice asked students who wished to participate to contact the senior investigator’s secretary or assistant for more details. Participation was voluntary and the investigators had no influence over the results of assessments, which might have encouraged participants to give socially desirable responses.

After a detailed explanation of the purpose of the study and its data collection procedures, recruits gave informed written consent to participate. Sixteen students agreed to participate and twelve of these provided the full dataset as described below. The four students who supplied incomplete data were excluded.

### Data collection

Participants recorded audio diaries of their clerkship experiences in Chinese. They were asked to make approximately 2–3 diary entries each week during the first 1–2 months of participation, totaling 12 entries. Each recording was to last around 30 min. The rationale for this sampling arrangement was to capture affective experiences that could best answer the research question. Each entry responded to these three questions:

1. What happened?
2. What did you feel/think/do?
3. How did this experience relate to your development as a doctor?

### Analysis

Diary entries were transcribed verbatim and entered into Atlas. Ti Version 6.0.15 (Atlas.ti GmbH, Berlin, Germany) for thematic analysis [23]. Data from six participants, translated into English, were included in a cross-cultural discourse analysis, which has already been published [9, 21]. The analysis reported here was a separate exercise, conducted in the original Mandarin rather than English, and using a different qualitative methodology, so it is an independent, though complementary, study.

Two authors independently read the verbatim transcripts of students’ diary entries. They open-coded text, identified themes, and synthesized an interpretive framework, constantly comparing this against participants’ original texts. They resolved disagreements by discussion with other members of the research team and reached consensus on a final coding. An author who cannot read or speak Mandarin (TD) helped the authors present their findings in English. The Chinese authors worked as a team to verify that this English language report accurately represented the original data. Text extracts are labeled numerically to maintain an audit trail back to the original data. The first two digits identify individual participants, the next two digits represent this student’s diary, and the number after the decimal identifies the specific episode.

## Results

The twelve students, five females and seven males aged 23–28, made 155 audio recordings (range 8–19, mean 13) between September 2014 and November 2014, each lasting between 15 and 55 min. Most diaries included learning experiences from 2 to 3 of the hospital’s specialty divisions.

### Vision of medical life

#### Expectations

Clerkship experiences made participants aware of their expectations about how life would be as a doctor, the specialties they would work in, and their future income and lifestyle. These expectations influenced participants’ affective reactions to clerkship experience and visions of medical life. The following extract describes how a participant’s expectations caused a negative reaction, which helped them form a vision of the future:

“I like going off-duty on time and saw residents (in my department) leaving the hospital at 5-6 pm today; however, life in the internal medicine department is quite different. How could a physician work more than 100 hours a week? Let my smart classmates choose that department. I couldn’t tolerate it. Poor sleep makes me depressed. Really!” (P1209.1).

A positive match between expectations and experiences tended to confirm participants’ visions, whilst mismatches might cause participants to reconsider their future specialty choices or even consider leaving medicine altogether.

#### Reality

Hard clinical realities influenced participants’ affective reactions and visions. In the next extract, the reality that aroused an affective response was patients not being satisfied with their care despite doctors working hard on a public holiday:

“On the National Day (a holiday in Taiwan) I saw that every senior doctor was present and, although they didn’t have to finish changing dressing wounds by 7:30 am as usual, some patients complained that a doctor had not yet changed their dressings.” (P0605.6).

Some participants accepted negative experiences more readily than others. If they found it hard to accept uncomfortable realities, participants’ visions of doctors’ relationships with patients changed:

“Before I was a medical student, if I saw a patient’s family member shouting ‘Where is the doctor?’ I would find a doctor … as soon as I could ‘ … however, now that I am in medical school … I ask myself what this person is doing, and why he is so unfriendly to doctors.” (P0411.3).

#### Values

Tensions between participants’ values when they entered medicine and their experiences aroused affective reactions. Observing a patient being reluctant to give his medical history to the attending physician and questioning and complaining about his treatment, rather than complying or expressing gratitude, made one participant angry. Tensions between participants’ values and patients’ high expectations and the risk of litigation aroused affective reactions too. The text that follows shows the heavy impact of such tensions on participants’ visions:

“Lawsuits … are a risk, too. No one wants to expose themselves to danger … especially after they have worked so hard for so long. It’s not fair. I think I have to know what I really want and, if I cannot change anything, leave for a better environment.” (P1101.2).

#### Preferences

Affectively positive experiences, like the one described next, helped participants form visions of their medical lives that fitted their personal preferences:

“I was called to assist at an operation .. and my senior asked me to cut the suture, which I did instantly in the right way. The experience stimulated my interest in hands-on activities and surgery.” (P0601.5).

### Personal resources

Participants’ personal resources influenced their affective responses.

#### Personality, character, and adaptability

One resource, which varied between participants and influenced their affective reactions, was the ability to understand other people’s feelings and needs and empathize with them. Here, a participant describes affective awareness:

“I met a patient with end stage liver cirrhosis. About half an hour before he died, the senior doctor finally obtained a do-not-resuscitate agreement. The atmosphere was a little creepy … Every doctor including the chief residents seemed so relieved. I felt really bad at that time.” (P0101.3).

Another resource was self-confidence. Being exposed to patients and their families, and being in an unfamiliar peer group, exhausted participants and elicited fear, distress, and an impulse to escape from the workplace. Some participants worried that patients or senior doctors would reject them. Starting on a new ward, for example, tested their affective resources:

“I am quite an introverted person. I like meeting people, but I … had too many new acquaintances! The pace was so fast and I just could not get used to it. I didn’t know how to address them. I didn’t know how to talk. I felt really exhausted.” (P0601.1).

Circumstances like these made unconfident participants anxious. More self-critical and less resilient participants were left with unresolved negative affects:

“Trying, failing, and having to ask someone to help made me feel guilty and useless; I doubted my suitability to be a doctor. I cried in the shower when I went home. My mom asked me why I was working so late. I asked her ‘Am I suitable to be a doctor?’” (P0602.4).

Perfectionism, self-centeredness, relationship anxiety, and defensiveness also affected participants’ affects. Being able to laugh at themselves, on the other hand, helped participants reflect on their weaknesses and transform clinical failures into determination to perform better. Participants who most rapidly became socially comfortable in clinical setting were best able to overcome the low moods that resulted from working under harsh conditions.

#### Past histories, health and fitness

Participants’ previous life experiences provided resources that influenced their affective reactions. Those who had led sheltered lives (perhaps because they came from wealthy families), and whose parents had not encouraged them to be independent, were ill-prepared for failures during clerkships and the lack of support when these happened:

“As a student who had always achieved flying colors … I was stuck with feelings of failure all night and gradually lost confidence. I tried to talk to patients .. but just couldn’t get rid of that bad feeling.” (P0602.6).

At the opposite extreme, participants who had, for example, participated in overseas exchange programs were less ready to acquiesce to peers and teachers, and accept poor quality educational experiences. This led to interpersonal conflicts and difficult affective reactions. Participants’ physical and psychosocial health also affected their affective reactions. They were afraid to take time away from work, and only did so if they were very sick.

#### Ways of learning

Participants’ preferred ways of learning affected their affective responses to changing from classroom to workplace education. Those who liked learning in a structured way now had to accept a lack of structure in clinical settings and do their book-learning during evenings after heavy days’ work, which they found exhausting. Some responded to this by doing as little clinical work as possible, not interacting with others, and disappearing to study privately. A participant described the tensions that resulted:

“I had to perk myself up very often or I would feel I was spending the whole day doing nothing. No new patients were admitted under my care, and I didn’t even do any physical examinations or procedures. I seemed just to be wasting time waiting around and feeling anxious.” (P0102.3).

Other students had more positive affective responses. Participants who were good at integrating and organizing small pieces of knowledge learned by providing clinical care. Participants who were not good at book learning enjoyed learning from discussion with others.

### Working resources

Another type of resource that influenced participants’ affects was the resources provided by their working environments, the organization of learning activities, training courses, and interactions with colleagues.

#### Working environments and their organization

The facilities, resources, working practices, and cultures of clinical workplaces influenced participants’ affects. The next extract describes how the social climate of a clinical department impacted positively on a participant:

“I like pediatrics because I like children and I feel that interpersonal relationships in the pediatrics department of our hospital are really nice.”(P0305.3).

Organizational hierarchies, on the other hand, were a cause of negative affects, which required participants to relate carefully to clinicians:

“I was very careful when I talked with my teachers. I heard lots of stories about medical students making their teachers angry by doing something wrong. I had to be careful.” (P0114.2).

Being given menial tasks caused difficult affects too:

“Our job was to get consent for surgery and make a note of the time we explained their biopsies to patients. We examined them and, again, wrote down the time, and … assessed their pain and so on. It was all boring paperwork. I didn’t know what I was doing.” (P0603.6).

#### Educational activities and interactions with clinicians

Since participants preferred to learn in different ways, educational activities affected their affects in different ways too. The next extract explains why a participant felt dissatisfied with their teaching.

“I didn’t ... learn much from ... psychiatry ... I was expecting practical lessons about how to distinguish depression from delusions, for example, and didn’t understand what they wanted me to learn and why they wasted so much time chatting. It seemed caring, but not professional lessons.” (P1201.3).

Teams with respectful cultures helped participants learn, which influenced their affects for the better, and vice versa. The following extract describes how a teacher praising their performance lifted a participant’s mood:

“When I presented a patient to my senior doctor, he told me a presentation should start by reporting the patient’s age and sex and then active problems, underlying diseases, chief complaints, and medications. I followed his advice when I presented to the attending physician … He praised me. I was very glad.” (P0301.4).

### Social resources

Supportive peers, family members, spouses, friends, and social organizations such as churches influenced participants’ affective reactions. These helped participants manage their reactions to, for example, tiredness and failure to perform clinical procedures:

“I want to become an intern with my classmates. As peers, we can laugh and cry together.” (P0604.2).

Family support helped participants ventilate pent-up emotions, as described in this extract:

“It was extremely frustrating. My mom said ‘You are more likely to fail if you get nervous. Practice makes perfect, so just do it.’” (P0602.7).

### Participation in practice

Once participants put on a white coat, hospital staff expected them to be competent. Patients and their families consciously or unconsciously transferred their own emotions to participants, whom they expected to give information senior physicians were unprepared to give. As well as patients’ negative emotions, participants reacted affectively to their own ineptitude.

#### Achievement

Rising to the challenges of medical work made participants more confident and helped them feel they were making progress. Positive feedback motivated them. Lack of achievement, on the other hand, demoralized participants. The following two extracts describe typical examples of participants’ ups and downs:

“When I was in the pediatric department, I explained the condition of their sick child all by myself, and I saw trust in family members’ eyes. I felt fulfilled because I think I offered help and also ‘leveled up’.” (P0102.4).

“I think my tracheal intubation skills … gradually improved. At least I began to feel familiar with the steps and stopped making mistakes.” (P0409.4).

#### Knowledge and experience

Clerkship experiences also highlighted participants’ lack of knowledge and skills:

“I didn’t study much so I didn’t know what to ask. It was a mess when I approached a new patient and it was messier when I tried to look for clinical signs.” (P0601.6).

Inexperience provoked negative affective reactions, as this extract describes:

“I intended to mask my nervousness. I came to the patient and explained with a trembling voice that I would draw blood from him. My hands were shaking as the needle punctured his skin.” (P0601.2).

Affective reactions became more positive as participants’ experience and skills increased.

#### Relating to patients and becoming professional

Interacting with sick patients and their families aroused strong affective reactions:

“The patient was much worse and the family were asked if resuscitation would be appropriate … it seemed like rubbing salt into their wounds and they did not know whether to say yes or no. I think the teacher was right. If I was the family member and you asked me if I would give up or not, and it was like I was forced to make the decision, I would feel guilty.” (P0101.2).

Patients’ and families’ harsh behavior could arouse strong negative reactions. Here, a participant describes fear of being humiliated:

“I saw the director being scolded in the ward. It was ridiculous. He was the most senior of us all; how could you humiliate him like that, and what about us juniors?” (P1101.3).

On the positive side, clinical experience could help participants explore their emerging professional identity, particularly regarding ethical practice and the need to respect others’ opinions. Here, a participant’s affective response was to think out loud about ethical aspects of a patient’s illness:

“To be terminal or not can be defined medically but a patient’s own feelings are more important.” (P0201.1).

Positive experience of identifying as professionals motivated participants to fulfill themselves.

## Discussion

Participants gave graphic accounts of how workplace experiences affected their mood, motivation, confidence, and attitudes. Experiences that aligned with and confirmed existing and/or future imagined identities evoked generally positive affects and experiences that were not aligned tended to provoke negative affective responses. Participants recruited personal and social resources to regulate the valence and strength of their identity emotions. When negative emotions were left unresolved, participants described a hardening of their attitudes towards patients, sowing the seeds of empathy decline.

Earlier research shows that our findings are characteristic of the Confucian culture in which this research was conducted, which differs from the Germanic culture against which earlier research compared it [9, 21]. Culturally-specific features of our participants’ reflective accounts were the ‘lively figured world with many different doctors, nurses, peers, patients, and relatives’ in which they were learning, the close relationship between participants’ identity construction and the moral and social values they held, and participants’ self-determination in relation to peers, patients, families, and senior doctors. Culturally specific features of participants’ learning environment include tensions with doctors that resulted from patients’ and their families’ values and expectations, a social expectation that hard work will be rewarded with gratitude, participants’ deference to supervising doctors’ seniority, the importance of proving oneself to others, and a work ethic that discouraged taking sick leave. That is not to say these cultural features are absolutely exclusive to Taiwan. Rather, that they are particularly strong sources of affective reactions in the Confucian culture that provided the context for this research.

There are limitations to the validity and transferability of this research. The English-language author who edited this article was not able to read the original data, and the Chinese authors were to some extent beholden to his preferred use of language. As in any research using a qualitative methodology, researchers’ individual subjectivities influenced the findings. This allowed our work to ‘represent complexity well’, [24] leaving readers to transfer insights to their own contexts and educational practices. The findings are to some degree specific to one year-group in medical school in one Confucian cultural context, and have not been compared with data from other contexts. One remaining limitation of this research is that its conceptualization of identity formation was drawn from Experience-based learning theory [14], whose roots are in social learning theory. Readers who conceptualise identity solely using individualistic, psychological models will, inescapably, find this a limitation of the work.

The evidence-base available to medical educators is strongly biased towards research from English language countries and Europe [14].This could easily result in cultures that dominate the evidence-base of our field imposing their cultural values on cultures that are less well represented in education research, if not in cultural richness and the sizes of their populations. This results in ‘educational cultural hegemony’ [22] or ‘Imperialism’ [25], both of which have been described as features of medical education.

## Conclusions

The main implication of our work to educational practice is that, to avoid hegemony or imperialism, educators should strive to be aware of the cultural norms that are prevalent in their place of work and develop critical consciousness of how individual students’ cultural backgrounds, expectations, and values influence their behaviors. The main implication for research is that research needs to be conducted in a wider range of cultures using culturally-sensitive research methodologies to guard against cultural imperialism in medical education. Only then will we be able to describe medical education, with any confidence, as a ‘global community’.

## Data Availability

The datasets used and analysed during this study are available from the corresponding author on reasonable request.
